# Addenbrooke's Hospital, Cambridge

**Published:** 1903-01-10

**Authors:** 


### ADDENBROOKE'S HOSPITAL, CAMBRIDGE.

At the Quarterly Court held at Addenbrooke's Hospital, Cambridge, the report of the Advisory Committee was brought up for discussion. One portion of it dealt with a communication received from the Charity Commissioners, enclosing a copy of a protest which had been received from 14 Governors, praying the Board to refuse their sanction to the scheme proposed for the government of the hospital. The Board intimated that they intended to treat this protest as an objection to the scheme, and to refer the matter to one of their inspectors for local inquiry. The Advisory Council also proposed to defer giving the detailed report of the finances of the hospital for the past year until a future meeting, owing to the fact that the certified copy of the accounts under the hands of the professional auditors had not been received. Many other matters were referred to, among them being the rules as to payment for medicines by the patients, which had recently been brought into operation. A good deal of discussion arose in regard to some parts of this report, but in the end it was adopted, showing that notwithstanding the objections raised in some quarters the advisory council possesses the confidence of the majority of those interested in the success of the hospital. Evidently the hospital is making progress, and the governors are gradually being led to accept the recommendations which were made when its affairs were so thoroughly inquired into some time ago.

